# Disseminated *Cunninghamella* spp. Endocarditis in a Beta-Thalassemia Patient after Asymptomatic COVID-19 Infection

**DOI:** 10.3390/diagnostics12030657

**Published:** 2022-03-08

**Authors:** Eliza Cinteza, Alin Nicolescu, Tatiana Ciomartan, Liana-Cătălina Gavriliu, Cristiana Voicu, Adelina Carabas, Monica Popescu, Irina Margarint

**Affiliations:** 1 Department of Pediatrics, “Carol Davila” University of Medicine and Pharmacy, 020021 Bucharest, Romania; elizacinteza@yahoo.com (E.C.); lianagavriliu@yahoo.com (L.-C.G.); 2 Department of Pediatric Cardiology, “Marie Curie” Emergency Children’s Hospital, 041451 Bucharest, Romania; nicolescu_a@yahoo.com (A.N.); cristiana.voicu@rez.umfcd.ro (C.V.); adelina.carabas@gmail.com (A.C.); doc.monica.popescu@gmail.com (M.P.); irina_margarint@yahoo.com (I.M.); 3 Department of Pediatrics, “Alessandrescu Rusescu” National Institute for Mother and Child Health, 020395 Bucharest, Romania

**Keywords:** endocarditis, *Cunninghamella* spp., mucormycosis, beta-thalassemia, iron overload, deferoxamine, asymptomatic COVID-19 infection

## Abstract

*Cunninghamella* spp. is a group of fungi belonging to the *Mucorales* order. Cases of fungal endocarditis are sporadic, but more frequent in immunocompromised patients. COVID-19 (SARS-CoV-2 Infection Disease 2019) infections, prematurity, deferoxamine treatment, iron overload, neutropenia, diabetes, and malignant hemopathies proved to be risk factors for mucormycosis. We present the case of a 7-year-old boy who was treated every three weeks with blood transfusion for major beta-thalassemia, receiving deferoxamine for secondary hemochromatosis. After two weeks with nonspecific respiratory and digestive symptoms, he was admitted for fever, followed by lower limb ischemia and neurological signs. Echocardiography revealed massive endocarditis affecting the mitral and tricuspid valves with embolization phenomena in the brain, lungs, kidney, spleen, and lower limbs. As a particular finding, IgG antibodies for COVID-19 were positive. Emergency cardiac surgery was performed. The mitral valve necessitated replacement with CarboMedics prosthesis. Unfortunately, the patient did not survive. *Cunninghamella* spp. was confirmed via the PCR analysis of vegetations. *Cunninghamella* endocarditis in the context of a systemic infection presented as an opportunistic infection affecting a child who had several risk factors. Mucormycosis is challenging to treat, with high mortality. Prophylactic treatment in beta-thalassemia patients with iron-chelator deprivation drugs, such as deferiprone, may help in preventing these particular fungal infections.

## 1. Introduction

Fungal infections in humans are rare, affecting 6/100,000 of the population. Mucormycosis represents the third-highest cause after candidiasis and aspergillosis, but the epidemiology of fungal infection varies among regions [1]. Among mucormycosis, *Cunninghamella* spp. infections represent 6.3–8% of them all [2,3]. Moreover, 8% of mucormycosis appears in children [4], usually associated with malignant hemopathies [5]. *Cunninghamella* spp. are fungi that belong to the *Mucorales* order [6]. Initially, a single species responsible for human infection, *Cunninghamella bertholletiae*, was described, but recently, *Cunninghamella arunalokei* was described as a new species infecting immunocompromised patients [7,8]. There are two features about mucormycosis which have an important influence on its morbidity and mortality: rapid progression with vascular invasion through thrombosis and necrosis, and it is found predominantly in immunocompromised patients [2]. The main risk factors include prematurity, age <12 months, iron overload, deferoxamine treatment, steroid use, diabetes, neutropenia, transplantation or hemopathies, intravenous drug use, renal impairment, malnutrition, broad-spectrum antibiotics or voriconazole prophylaxis, and COVID-19 infection (or post-COVID-19 infection) [2,3,9,10,11,12,13,14]. *Cunninghamella bertholletiae* is more frequently associated with malignant hemopathies with neutropenia (57%), nonmalignant hematologic conditions requiring chronic transfusions, diabetes mellitus, transplant receipt, and asplenia [7].

Many reports discuss the role of high ferritin levels associated with increased iron concentrations leading to the release of reactive oxygen species that might damage the tissues. This situation may apply to COVID-19 infection, thalassemia, malignant hemopathies, and many other diseases [9]. In children with beta-thalassemia, at least three risk factors are found (iron overload, deferoxamine usage, and regular intravenous transfusion); therefore, they have a higher risk for these infections. Reports for mucormycosis in thalassemia patients are frequent, but other infections may be specific, too, such as Yersinia enterocolitica or coinfections with *Pneumocystis jirovecii* (previously *Pneumocystis carinii*), HIV, and cytomegalovirus [6,7,15,16,17].

In adults, mucormycosis may present as rhino–orbital–cerebral, in the lungs, as gastrointestinal or cutaneous infection, or as a disseminated infection [5]. The latter is the most severe form, with a 96% mortality rate. In children, gastrointestinal infections are frequent, with a rate of 54%, and even more frequent in neonates [18]. Children may also have cutaneous involvement (36%). In older children, the incidence of sinopulmonary and rhino–cerebral infections is similar to adults [18]. The highest mortality rates were reported in neonates, disseminated forms, and young age (<1 year) [11,18]. Although the cardiovascular system is affected by definition, the mechanism of invasion being thrombosis and infarction, cases of cardiac mucormycosis are sporadic, many of them being included in disseminated forms, which means the involvement of at least two non-contiguous organs. Cardiac mucormycosis may be endocardial (the most frequent form), myocardial, pericardial, or mixed [3,19,20,21,22,23,24,25,26,27,28,29,30]. Mucormycosis is a rare cause of endocarditis in intravenous drug users. Additionally, coronary embolization may appear with extended myocardial infarction or heart block and arrhythmias [31].

## 2. Case Report

We present the case of a 7-year-old boy who was treated every three weeks with blood transfusions for major beta-thalassemia, receiving deferoxamine for secondary hemochromatosis. After an uneventful pregnancy, he was born at term (gestational age 39 weeks, birthweight 3950 g, APGAR score 9). Both his parents were known to have minor thalassemia. At the age of 9 months, he was diagnosed with major beta-thalassemia, and he received blood transfusions every three weeks. At five years of age, he developed sepsis with Yersinia enterocolitica.

The present illness had an insidious onset, with coryza and rhinorrhea, followed by watery stools for five days. After ten days of nonspecific respiratory and digestive symptoms, he presented at the emergency room where he was evaluated. Blood tests and chest X-rays were regular, except for anemia, which was associated with his major beta-thalassemia. Subsequently, he was admitted to another hospital for persistent fever (39 °C), followed by the reappearance of watery stools. Then, 24–48 h after admission, he presented asymmetrical temperature at the lower limbs, one being colder than the other, and intermittent neurological signs, such as obnubilation and delirium.

Clinical examination showed a febrile 7-year-old male (38.7 °C, axillary), with a height of 131 cm, a weight of 25 kg, a discrete erythematous maculopapular rash on the ventral part of the foot, serious general condition, a respiratory rate of 55–60/min, oxygen saturation at 85% in room air and with 99% oxygen 4 L/min by facial mask, costal retractions, bilateral crackles at pulmonary auscultation, a systolic murmur II/VI, a heart rate of 140/min, blood pressure at 101/53 mmHg, diuresis present, moderate hepatosplenomegaly, diffusely painful abdomen on palpation, and altered consciousness (Glasgow Coma Scale 14).

A complete blood count revealed white blood cells at 13.690/mm^3^, lymphocytes at 4.2% (570/mm^3^), neutrophils at 85.2% (11,670/mm^3^), monocytes at 10.1% (1300/mm^3^), red blood cells at 2,890,000, hemoglobin at 8.3 g/dL, hematocrit at 23.2%, and platelets at 43.000/mm^3^.

A peripheral blood smear showed neutrophils with hypertoxic granulations and vacuoles in the cytoplasm, microcytosis, anisocytosis, moderate hypochromia, and thrombocytopenia.

Serum electrolytes were quasi-normal (Na 135 mmol/L, K 4.6 mmol/L, Ca 1.16 mmol/L), with high serum lactate (5.5 mmol/L). IgA antibodies for Yersinia enterocolitica were positive, 4.851 (N < 0.8). A nasopharyngeal sample for SARS-CoV-2 (RT-PCR) was taken 48 h after onset of symptoms, and the result was negative, but IgG antibodies were positive. IgG were present because of a possible infection two weeks prior to the first admission to the hospital when the child had symptoms of upper respiratory tract infection and diarrhea. The patient was not vaccinated for SARS-CoV-2.

Cerebrospinal fluid (CSF) was mildly opalescent, with polymorphonuclears 224/mm^3^, normal protein and glucose levels (25.6 mg/dL—N 15–45, and 60 mg/dL—N 40–75, respectively), negative C-Reactive protein, and high lactate at 3.16 mmol/L (N1.1–2.4). Blood glucose level was 119 g/dL.

**Figure 1:**
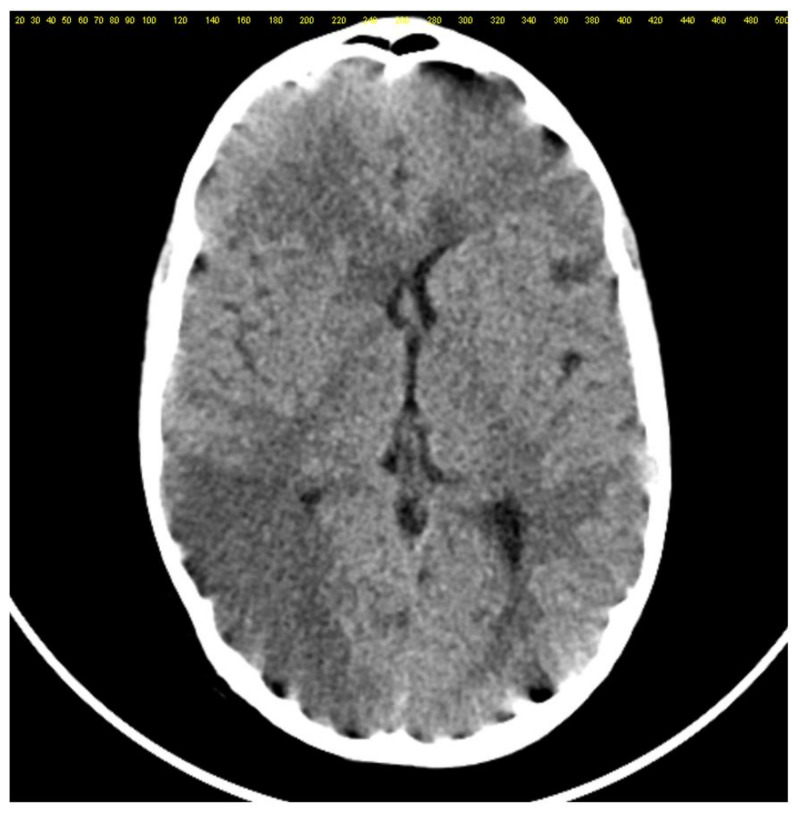
AngioCT modifications with multiple areas of hypodensity in the right parietal lobe and left pericallosal cerebral hypodensity associated with hyperdensity in the surrounding area.

A cerebral angioCT showed multiple areas of hypodensity in the right parietal lobe of 35/15/17 mm and a left pericallosal cerebral hypodensity of 35/14/15 mm with hyperdensity in the surrounding area (Figure 1).

**Figure 2:**
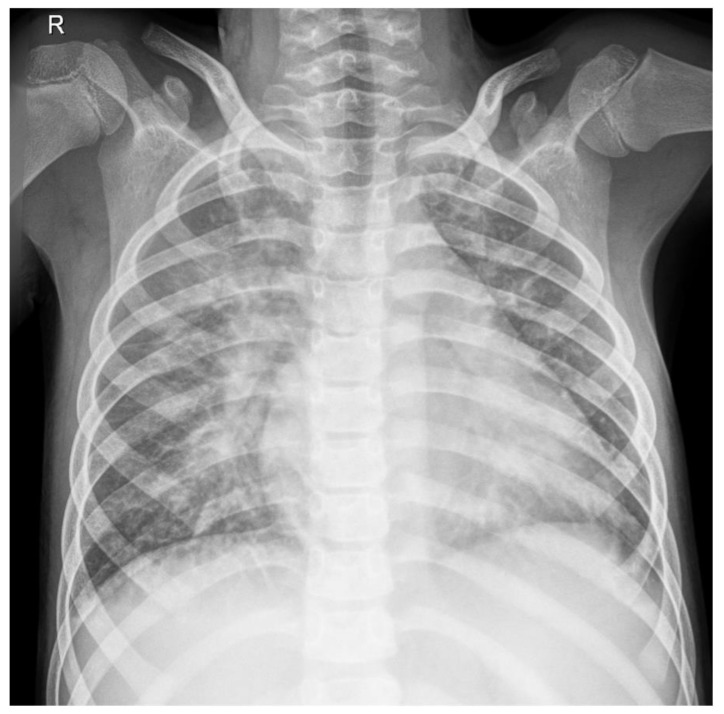
Chest X-ray showing veiling of both costo-diaphragmatic recesses and numerous reticulonodular opacities, with erased contour and tendency to confluence, diffusely distributed in both lung fields. R, right.

Chest X-ray findings included veiling of both costo-diaphragmatic recesses and numerous reticulonodular opacities, with erased contour and tendency to confluence, diffusely distributed in both lung fields (Figure 2).

**Figure 3:**
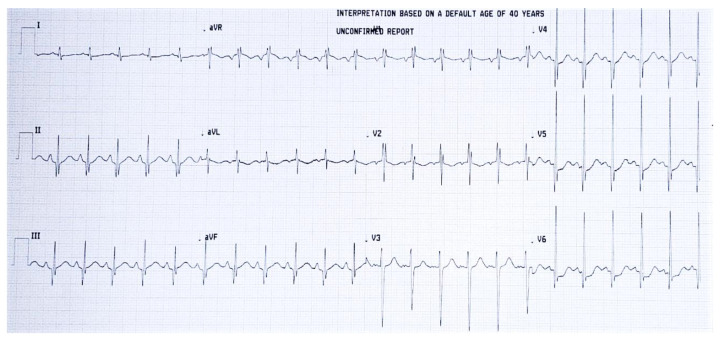
ECG showing pathological Q waves in DII, DIII, aVF, V4–V6, 2 mm depression ST segment in V5–V6, negative T waves in DI, aVL, suggestive of ischemia, lesion, and necrosis.

An electrocardiogram revealed pathological Q waves in DII, DIII, aVF, V4–V6, 2 mm depression ST segment in V5–V6, negative T waves in DI, aVL, suggestive of ischemia, lesion, and necrosis (Figure 3).

**Figure 4:**
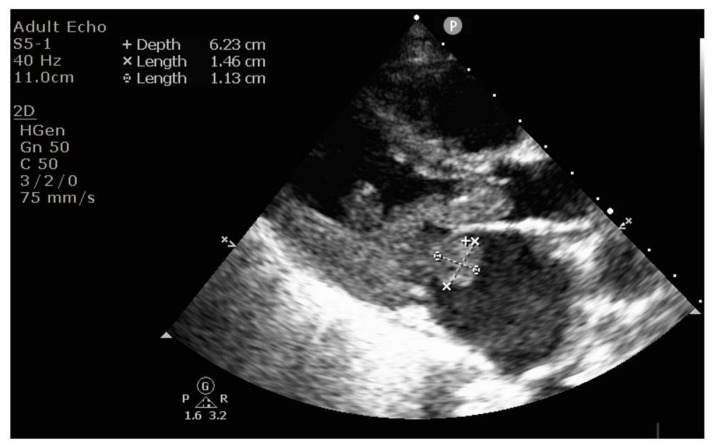
Mitral endocarditis below and above the mitral leaflets, on both atrial and ventricular sides.

**Figure 5:**
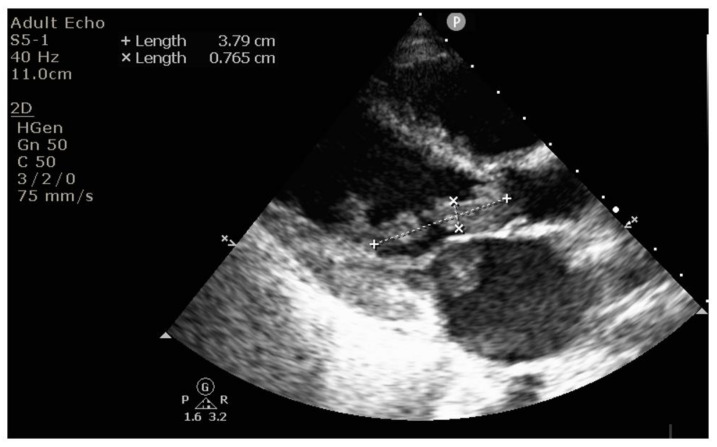
Long vegetation attached to the mitral valve below the valve, directed towards the left ventricle ejection tract, measuring 3.8 × 0.7 cm.

**Figure 6:**
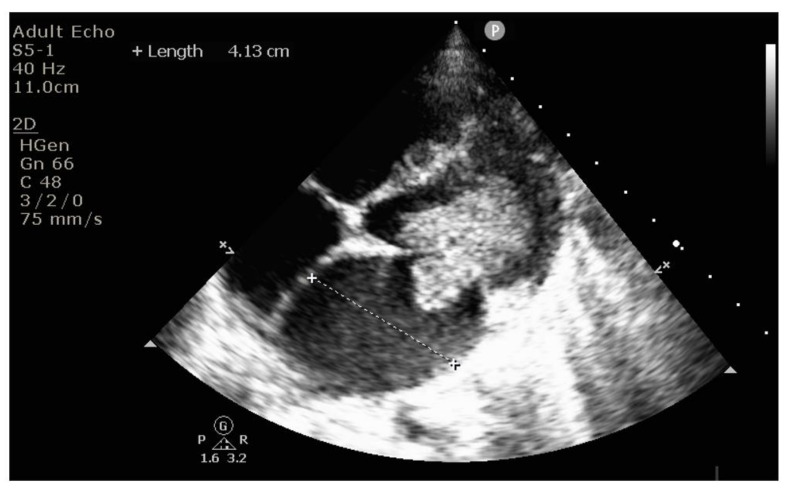
Dilated left atrium due to obstruction effect similar to mitral stenosis, generated by the mitral vegetation blocking the flow towards the ventricle.

**Figure 7:**
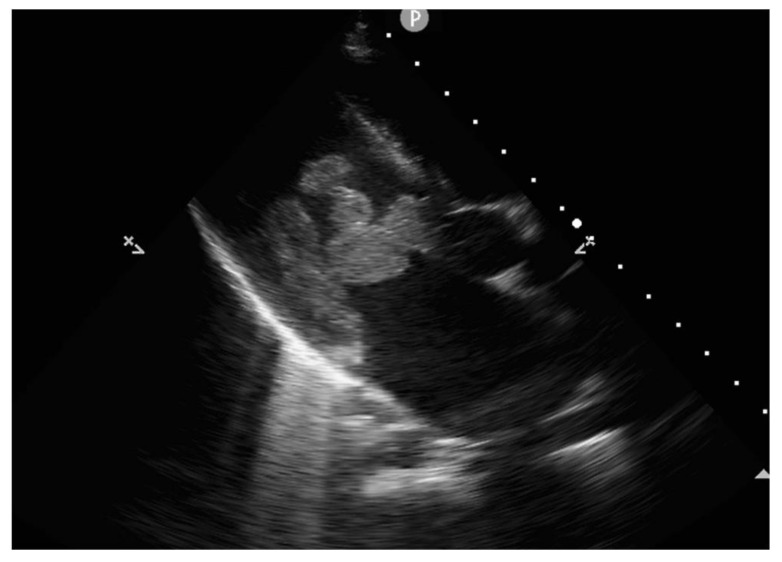
Echocardiography shows numerous vegetations at the mitral level, of different shapes and dimensions, obstructing the left ventricular inflow, with high mobility especially of the most distal one.

**Figure 8:**
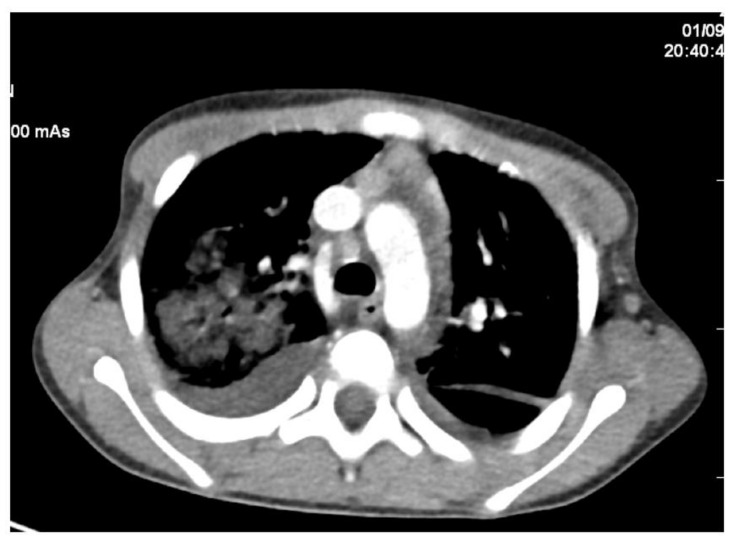
Thoracal angioCT shows important consolidation of the right lung, posterior right pleural liquid.

**Figure 9:**
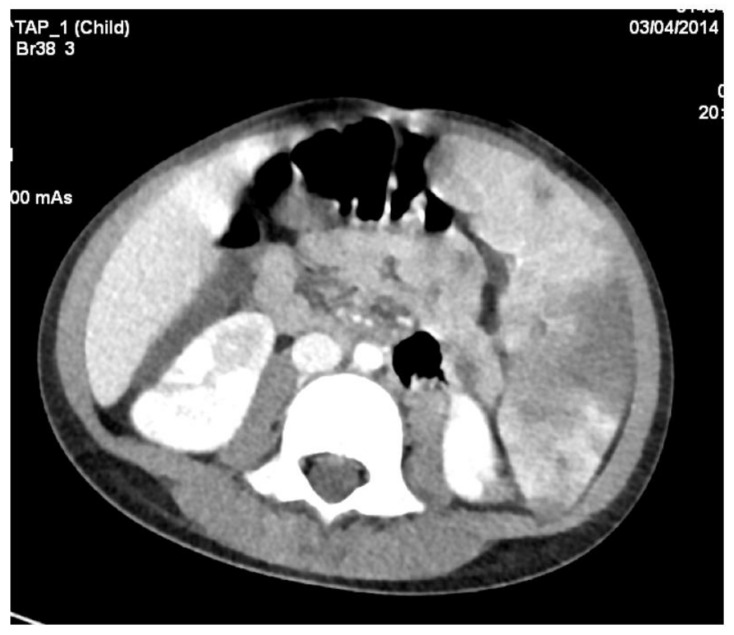
Abdominal angioCT shows multiple infarctions at the kidneys and spleen level.

Echocardiography revealed massive endocarditis severely affecting the mitral valve (Figure 4, Figure 5, Figure 6 and Figure 7) and also the tricuspid valve (mildly), with embolization phenomena in the brain, lung, kidney, spleen, and lower limbs (Figure 8 and Figure 9). Multiple mitral and tricuspid valve vegetations were identified. The largest mitral vegetations measured at 11/14 mm, 15/18 mm, and 8/38 mm, the last protruding through the aortic valve. Hemodynamic effects of severe functional mitral stenosis were present. The medical history revealed that the last cardiac ultrasound was performed two years ago, one month after the Yersinia infectious episode, and the result was normal.

**Figure 10:**
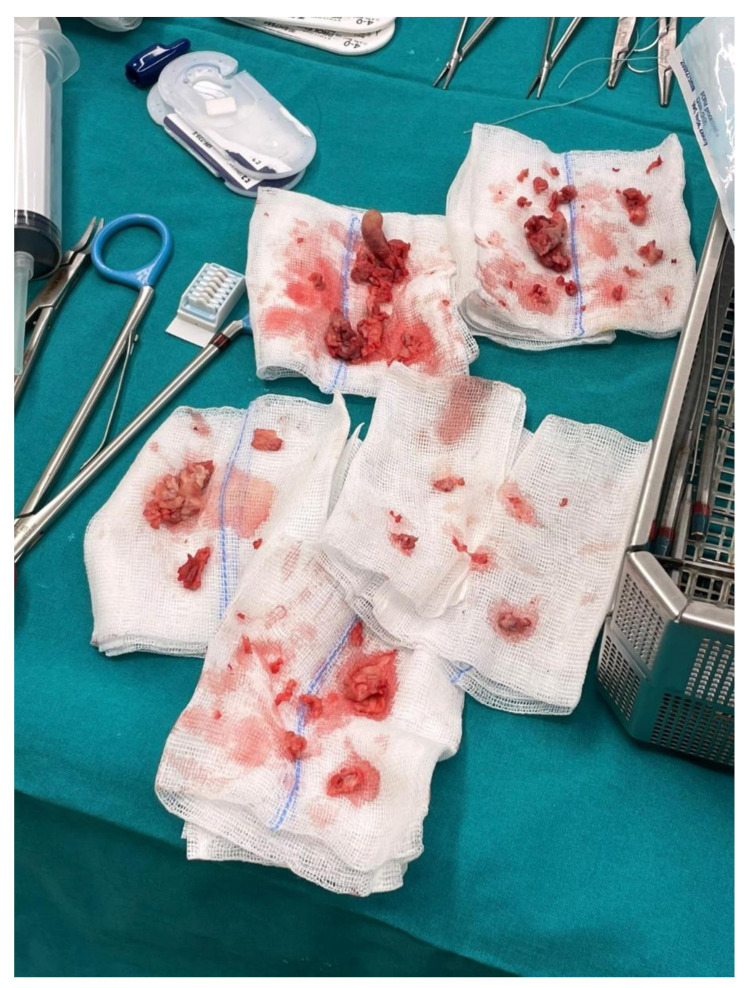
Anatomopathological specimens extracted from both mitral and tricuspid valves represent multiple vegetations of different dimensions.

Emergency cardiac surgery for mitral and tricuspid valves was performed. An extensive cardiac surgical intervention was initiated with the excision of massive vegetations of the mitral valve (Figure 10) and shaving of the tricuspid valve. Mitral valve replacement was required and it was performed with CarboMedics prosthesis. Unfortunately, the patient did not survive in the following hours, manifesting severe hypotension on the background of severe cardiac dysfunction and vasoplegic shock in the context of fungal sepsis, despite the maximum inotropic and vasoactive treatment. The cultures performed from the vegetation yielded filamentous fungi, which presented macroscopic morphological characteristics and microscopic characteristics of the order Mucorales, *Cunninghamella* spp. Additionally, a polymerase chain reaction (PCR) performed from the vegetations revealed *Cunninghamella* spp. as the etiology of the infection.

## 3. Discussion

*Cunninghamella* spp. is part of the *Zygomycetes* or *Mucorales* order. This order also includes *Rhizopus, Mucor*, *Rhizomucor*, *Absidia*, *Apophysomyces*, *Syncephalastrum*, *Saksenaea*, *Cokeromyces*, *Entomophthora*, *Conidiobolus*, and *Basidiobolus* [6]. Although the disseminated form of *Cunninghamella* infection was described in 1958 by Hutter, very few cases were reported until recently (around 200 cases up to 2009) [11]. COVID-19 increased the number of cases due to immunosuppression associated with COVID-19 infection and steroidal or immunosuppressive medication [12]. Immunocompromising conditions should be well known because, although the infections are rare, this group of patients is severely affected. Besides the malignant or non-malignant hemopathies group, there are patients receiving immunosuppressive medication, including steroids, diabetic patients, and recently the COVID-19 infection or past COVID-19 infection.

Our patient had many risk factors for mucormycosis, being chronically transfused for major beta-thalassemia, with iron overload, being treated with deferoxamine, and having an asymptomatic past COVID-19 infection. The asymptomatic form of past COVID-19 infection does not necessarily guarantee mild consequences on the immune system, as many pediatric inflammatory multisystem syndromes (PIMS) appeared in asymptomatic children. Our patient had predisposing factors for generalized disseminated infection, such as deferoxamine use. Deferoxamine binds the iron molecules and generates an iron-enriched system that serves as a siderophore for these fungi, which use the iron for rapid growth [6]. Iron overload may also favor the growth of the fungi by compromising the ability of the phagocytes to perform their antifungal role [32]. Iron-chelators other than deferoxamine (deferasirox or deferiprone) did not show a xenosiderophore activity, and they must be used as part of the treatment in patients with beta-thalassemia who need iron-chelator therapy [33].

*Cunninghamella* spp. is more frequently found in both localized (pulmonary and rhino-sinusal) and disseminated forms [7,10], while in children, the gastrointestinal forms of the disease are more frequent [18]. Our patient progressed from a mild form of upper respiratory tract infection (URTI) to persistent fever and prolonged diarrhea and ultimately a severe disseminated infection within two weeks. As the patient was known with a normal cardiac structure of the heart from previous echocardiography examination, it is surprising that the cardiac valves (mitral and tricuspid) were implicated so severely, with massive vegetations on both valves, predominantly on the mitral valve, generating severe mitral stenosis responsible for pulmonary edema. Cardiac structures might have been affected by contiguity from pulmonary alveoli, as pneumonia could have complicated the URTI. In our case, a CT scan and chest X-ray showed images of bronchopneumonia. Additionally, ingestion of the spores might have determined the gastrointestinal form manifested with abdominal pain, vomiting, and diarrhea.

15% of mucormycosis cases may be disseminated forms. These forms are associated with prematurity, iron overload, chronic treatment with deferoxamine, and immunodeficiency. Once disseminated, the prognosis changes to the worst, the mortality rate being 96%, especially for the *Cunninghamella* infection, which also has a terrible prognosis. This was the case of our patient who had hemochromatosis, iron overloading, and secondary to major beta-thalassemia, having been treated for several years with deferoxamine. The asymptomatic COVID-19 infection might have had induced secondary immunodeficiency [6,18].

There are no suggestive clinical signs for mucormycosis. With specific laboratory investigations that might suspect the diagnosis, the halo sign may appear in mucormycosis as in aspergillosis, but less specific, or in other fungal infections [34]. Galactomannan and glucan detections are used for aspergillosis diagnosis [3], but they are not valuable for mucormycosis. Polymerase chain reaction (PCR) from tissue or fungal cultures obtained in reference laboratories or histopathological diagnosis are the best tools to diagnose mucormycosis [18,35]. Recent techniques of PCR might identify *Absidia*, *Apophysomyces*, *Cunninghamella*, *Mucor*, *Rhizopus*, and *Saksenaea* [36]. Once the diagnosis is made, quantitative PCR (qPCR) may be used within seven days after treatment initiation to monitor the response to liposomal–amphotericin B. A negative qPCR after seven days of treatment is associated with an 85% lower 30-day mortality rate [37]. Pulmonary findings might be similar to pulmonary aspergillosis, with infiltration, consolidation, nodules, cavitations, atelectasis, effusion, posterior band thickening, hilar or mediastinal adenopathy, or normal appearance [5].

Prognosis is poor for mucormycosis, with death rates between 45 and 65% in localized infections and 96% in disseminated infections [4]. Only 3% of the patients with mucormycosis that did not receive specific treatment survived [6].

Our patient suffered cardiac surgery for the mitral and tricuspid vegetations already disseminated in many organs and systems. The short time elapsed from the moment of the endocarditis diagnosing precluded the initiation of antifungal therapy, as emergent cardiac surgery was required for the severe hemodynamic effects of functional severe mitral stenosis due to multiple vegetations. The only therapeutical possibilities are the surgical removal of the affected tissue and antifungal treatment with amphotericin B, posaconazole, or isavuconazole. Factors that could improve prognosis include removing the immunosuppressives and excluding predisposing medical factors. Furthermore, prophylactic use of deferasirox or deferiprone, which has no potentiating role on Mucorales as deferoxamine, could reduce the risk of mucormycosis [3,15].

## 4. Conclusions

*Cunninghamella* spp. infections have severe outcomes, with dissemination starting from initial pulmonary localization. The initial symptoms and clinical signs might be mild, but the progress is rapid and dramatic. Fungal infections are difficult to treat, and mucormycosis, especially the *Cunnighamella* spp. in disseminated form, has a very high mortality. Prophylactic treatment in beta-thalassemia patients with iron-chelators that do not act as iron-providers for fungus, but as iron-depriving drugs, such as deferiprone or deferasirox, represents an option that can improve outcomes in severe fungal infections.

## Data Availability

Not applicable.
